# Novel Gene Therapy Viral Vector Using Non-Oncogenic Lymphotropic Herpesvirus

**DOI:** 10.1371/journal.pone.0056027

**Published:** 2013-02-11

**Authors:** Akihiro Shimizu, Nobuyuki Kobayashi, Kazuya Shimada, Kuniaki Oura, Tadao Tanaka, Aikou Okamoto, Kazuhiro Kondo

**Affiliations:** 1 Department of Virology, The Jikei University School of Medicine, Tokyo, Japan; 2 Department of Obstetrics and Gynecology, The Jikei University School of Medicine, Tokyo, Japan; Osaka University Graduate School of Medicine, Japan

## Abstract

Despite the use of retroviral vectors, efficiently introducing target genes into immunocytes such as T cells is difficult. In addition, retroviral vectors carry risks associated with the oncogenicity of the native virus and the potential for introducing malignancy in recipients due to genetic carryover from immortalized cells used during vector production. To address these issues, we have established a new virus vector that is based on human herpesvirus 6 (HHV-6), a non-oncogenic lymphotropic herpesvirus that infects CD4^+^ T cells, macrophages, and dendritic cells. In the present study, we have altered the cell specificity of the resulting recombinant HHV-6 by knocking out the U2–U8 genes. The resulting virus proliferated only in activated cord blood cells and not in peripheral blood cells. Umbilical cord blood cells produced replication-defective recombinant virus in sufficiently high titer to omit the use of immortalized cells during vector production. HHV-6 vectors led to high rates (>90%) of gene transduction in both CD4^+^ and CD8^+^ T cells. These viruses showed low-level replication of viral DNA that supported greater expression of the induced genes than that of other methods but that was insufficient to support the production of replication-competent virus. Furthermore, HHV-6 vectors containing short hairpin RNAs against CD4 and HIV Gag remarkably inhibited the production of these proteins and HIV particles. Here we demonstrate the utility of HHV-6 as a new non-carcinogenic viral vector for immunologic diseases and immunotherapy.

## Introduction

Gene introduction into T cells is a very useful technique for gene therapy of HIV infection and the immunotherapy of fatal diseases including cancer. This method currently relies on vectors derived from members of the lentivirus family of retroviruses to introduce genes into T cells [1]. A major advantage of retroviral vectors is the high efficiency with which they introduce genes into target cells. However, the pathogenicity of the native virus has long caused unease regarding the use of viral vectors. In particular, oncogenicity is a characteristic of wild-type retroviruses [2]; another risk factor is the potential recombination of retroviral vectors with endogenous retroviruses in the host to yield replication-competent virus [3].

Adeno-associated virus (AAV) vectors have been developed to improve the safety of viral vectors and their transduction into hematologic cells [4]. However, the packaging capacity of recombinant AAV is restricted to approximately 5 kb because of the small size of the viral genome [5] [6]. Furthermore, because (unlike wild-type AAV) recombinant AAV vectors can integrate randomly into host chromosomes [7] [8], recombinant AAV vectors cannot be guaranteed to be free from carcinogenic effects.

Another risk factor for the induction of neoplasia in recipients results from use of cell lines during vector production. For the production of nonproliferating virus, the use of a cell line that expresses a deficit gene is essential, but some cell lines are not completely free of carcinogenic potential. Even well-known cell lines such as HEK293T cells are not free of the risks of tumor induction due to the cell line itself or to the impaired genetic stability of the retrovirus vector [9] [10]. Extensive characterization is required to address the suitability of potentially neoplastic cell substrates for viral vector manufacture [11].

Here, we have manipulated human herpesvirus 6 (HHV-6) to create a viral vector that overcomes these problems. Because herpesviruses are large double-stranded DNA viruses, they have the great advantages of being able to package and introduce large DNAs into target cells. There are eight types of human herpesvirus, and the target cells for infection and gene transduction differ accordingly. For example the representative herpesvirus herpes simplex virus type 1 (HSV-1) infects nerve cells, and viral vectors based on HSV-1 are neurotropic vectors that can introduce genes into neurons. Lymphotropic herpesvirus vectors based on Epstein–Barr virus transfect B cells, and those based on herpesvirus saimiri have been developed for T cells [12] [13]. However, all of these herpesviruses, like retroviruses, are oncogenic viruses and therefore are associated with the same disadvantages regarding their clinical use.

In contrast, HHV-6 is a low-pathogenicity, non-carcinogenic herpesvirus that infects immune cells including T cells, macrophages, and dendritic cells [14] [15]. This virus causes exanthema subitum, a mild disease that affects immunocompetent persons during childhood [16]. Members of the β-herpesvirus subfamily to which HHV-6 belongs share the US22 family of genes, which controls the host cell specificity of the virus. Removal of this gene cluster may render HHV-6 growth-defective in certain kinds of cells [17]. In the present study, we deleted several US22 family genes to create a recombinant HHV-6 that was growth-competent only in stimulated umbilical cord blood cells and was growth-defective in the T cells that usually support HHV-6 proliferation. Moreover, our recombinant HHV-6 showed high transduction efficiency into both CD4^+^ T cells and CD8^+^ T cells and subsequent strong transgene expression in the infected cells.

## Materials and Methods

### Viruses and cells

Wild-type HHV-6 (wtHHV-6; HHV-6 variant B [HHV-6B] strain HST) and its recombinant viruses were propagated in anti-CD3 antibody-stimulated human umbilical cord blood mononuclear cells (CBMCs) until more than 90% of the cells showed cytopathic effects. Cultured cells then were frozen at −80°C, thawed twice, and centrifuged at 2000× *g* for 5 min. The supernatant fraction was stored at −80°C as a cell-free virus stock.

For the titration of recombinant viruses, MT4 cells purchased from JCRB Cell Bank (Saito, Osaka, Japan) were infected and cells positive for enhanced green fluorescent protein (EGFP) were counted 2 d after infection. To titer wtHHV-6, MT4 cells were infected and underwent an indirect immunofluorescent assay (IFA) technique using anti-HHV-6 monoclonal antibody as described previously [18]. Virus titer was indicated as the number of focus-forming units per milliliter (FFU/ml). wtHHV-6 and recombinant HHV-6 were analyzed by using stimulated peripheral blood mononuclear cells (PBMCs) and CBMCs. We purchased CBMCs from RIKEN BioResource Center (Tsukuba, Ibaraki, Japan). The ethical committee of the RIKEN Tsukuba Institute approved the use of human umbilical cord blood before the study was initiated. We also obtained CBMCs, PBMCs and human serum from The Jikei University Hospital. Informed and written consent were obtained from the blood donors, and the protocol was approved by the Ethics Committee of The Jikei University School of Medicine.

PBMCs and CBMCs were separated by using Ficoll-Conray density gradient centrifugation [19], cultured in RPMI 1640 medium (Invitrogen, USA) supplemented with 10% heat-inactivated fetal bovine serum (FBS; Invitrogen) and 10 µg/ml IL2 (ProLEUKIN; Chiron Corp, USA), and activated with 5 µg/ml phytohemagglutinin (PHA; J-Oil Mills, Japan) or on plates coated with 1 µg/ml anti-CD3 antibody (orthoclone OKT3; Janssen). To infect PBMCs or CBMCs with viruses, cells were suspended in 1 ml of virus solution at a multiplicity of infection (MOI) of 0.5 to 2.0 and were spun at 1500× *g* for 30 min at 37°C for adsorption [20].

### Vector plasmids

To create the parental vector plasmid, we first replaced the U2–U8 gene cluster of HHV-6B HST with the selectable marker sequence EGFP-IRES-LNGFR-Puro; this marker sequence contains the gene for EGFP under the control of the human cytomegalovirus (HCMV) major immediate-early enhancer promoter (MIEP) and the gene for low-affinity nerve growth factor receptor (LNGFR) linked with EGFP by using an internal ribosomal entry site (IRES) and the puromycin-resistance gene (Puro) under control of the simian virus 40 (SV40) early promoter. To this end, the U2 gene of HHV-6B was PCR-amplified by using primers U2 Afl II and U2 Xba I, and the U8 gene was amplified with primers U8 EcoR I and U8 BamH I; primers used in this study are listed in Table S1. In particular, primer U2 Afl II (nucleotides 10184 to 10216 of the HHV-6B genome) binds within the promoter sequence of the U2 open reading frame (ORF), and primer U8 BamH I (coordinates 16547 to 16581) was designed to complement a region between the stop codon and the poly(A) signal of the U8 ORF. The amplified products then were inserted into each end of the EGFP-IRES LNGFR-Puro cassette (pU2-U8 LEP).

The short hairpin RNA (shRNA) inserts against CD4 and HIV p24 Gag that we generated each contained a sense strand and antisense strand, and these were linked by a standard GTGTGCTGTCC loop structure that is used in mammalian cells [21]. We created three A-to-G or C-to-T mutations in the sense strand, which not only prevented unwanted mutations in *Escherichia coli* cells but also enhanced the silencing activity [22]. We used a U6 promoter to drive our shRNA constructs because it is known to work in mammalian cells [23].

To create the shRNA inserts, sense and antisense oligonucleotides containing the terminator sequence (TTTTT) and *Bam*HI and *Hin*dIII sites at their 5' and 3' ends, respectively, were synthesized. The targeted sequence in CD4 was 5′-GGCTAGATGATTGATTACCAAGT-3′ and the targeted sequence in HIV p24 Gag was 5′-GATTGTACTGAGAGACAGGCT-3′. The resulting complete sequence of the shCD4 sense strand was 5′-gatccccGGCTgGATGATTGgTTACCgAGTgtgtgctgtccACTTGGTAATCAATCATCTAGCCttttttgaaa-3′, and that for the shGag sense strand was

5′-gatccGATTGTAtTGAGAGAtAGGtTgtgtgctgtccAGCCTGTCTCTCAGTACAATCttttta-3′ (lowercase letters indicate restriction enzyme sites, artificial mutations [A-to-G or C-to-T], loop structure, and the terminator sequence). The complementary oligonucleotides for each shRNA insert were synthesized, annealed, and ligated into the *Hin*dIII and *Bam*HI sites in the downstream region of the PolIII U6 promoter of pSilencer 2.1-U6 Puro (Ambion; Life Technologies, USA). pHSG 396 (Takara Bio, Japan) was cut with *Hin*dIII and *Eco*RI to replace its multiple cloning site with a 35-bp oligonucleotide adaptor containing *Cla*I, *Eco*RI, and *Hin*dIII restriction sites (5′-AGCTCATCGATGAATTCCGCGAAGCTTCATCGATT-3′). The U6-shRNA cassette was excised from pSilencer 2.1-U6 Puro containing the shRNA by using *Hin*dII and *Eco*RI and ligated into the *Hin*dIII-*Eco*RI site of the modified pHSG 396.

To construct the pU2-U8 shRNA plasmids (pU2-U8 U6 shCD4, and pU2-U8 U6 shGag) for recombination with HHV-6, the U6-shRNA cassette was excised by using *Cla*I and inserted into *Cla*I restriction site located between the HCMV IE promoter and SV40 early promoter of pU2-U8 LEP. In addition, each shRNA was ligated into the *Hin*dIII-*Bam*HI site of pSilencer 2.1-U6 Puro to use as a control plasmid.

Self-inactivating (SIN) lentivirus vector plasmids (pCAG-HIVgp, pCMV-VSV-G-RSV-Rev, CS-CDF-CG-PRE) were provided by Dr. Hiroyuki Miyoshi (RIKEN BioResource Center, Ibaraki, Japan), and luciferase plasmid (pGL3 vector) was purchased from Promega.

### Generation of recombinant HHV-6 (H6R28LEP)

CBMCs were stimulated with PHA for 2 d and then infected with wtHHV-6 by using the centrifuge method [20].The cloned plasmids pU2-U8 LEP, pU2-U8 U6 shCD4, and pU2-U8 U6 shGag were transfected into HHV-6-infected CBMCs by using a Nucleofector electroporator (Amaxa; Lonza, Switzerland) according to the manufacturer's protocol. Briefly, 5×10^6^ cells were mixed with 1 µg of the cloned plasmid and 100 µl of Human T cell Nucleofector solution, and electroporated with the Nucleofector using program T-20.

To enrich the titers of recombinant virus stocks, infected CBMCs were mixed with stimulated CBMCs, cultured for 1 d, treated with 1 µg/ml puromycin for 1 d; transfected cells were selected by using the MACS system (Miltenyi Biotec, Germany) according to the manufacturer's instructions. Briefly, 1×10^7^ cells were suspended in 70 µl buffer (PBS supplemented with 0.5% FBS and 2 mM EDTA), mixed with 20 µl MACSelect LNGFR MicroBeads, incubated for 15 min at 4°C, washed, resuspended in 1 ml of the buffer, and applied to the LS column, which was placed in the MidiMACS magnetic separator. The column was washed three times and removed from the magnetic separator. The selected CBMCs were mixed with stimulated CBMCs and cultured for 3 d. Culture supernatants of the infected CBMCs were used as new virus stocks after the freeze–thaw cycles described earlier. This selection procedure was repeated 5 times, and we successfully obtained highly pure high-titer recombinant virus (H6R28LEP) within 1 month.

### Flow cytometric analysis

Stimulated PBMCs, unstimulated PBMCs, and unstimulated CBMCs were all infected with H6R28LEP (MOI, 2). Fluorescent activated cell sorting (FACS) analysis was performed to assess the percentage of infected PBMCs at 4 d after infection. Stimulated PBMCs were infected with H6R28LEP or H6R28LEP shCD4 (MOI, 2). FACS was performed to determine CD4 expression levels on the surface of PBMCs at 4, 6, 8, and 10 d after infection. Infected PBMCs were stained with APC-tagged anti-human CD4 antibodies, APC-tagged anti-human CD8 antibodies, or APC-tagged anti-human CD19 antibodies, whereas infected CBMCs were stained with APC-tagged anti-human CD34 antibodies (all from BD Biosciences, USA). Transduction efficiency was determined by assaying for EGFP expression. FACS analysis was performed on a BD FACSCalibur instrument using BD CELLQuest software for analysis. Results were verified by performing at least three independent experiments.

### Virus infection and plasmid transfection

PBMCs (5×10^6^ cells) were infected (MOI, 0.5 to 2.0) with each recombinant HHV-6 or wtHHV-6 by using the centrifuge method. For siRNA expression, 5×10^6^ PBMCs were transduced with 1 µg of pSilencer 2.1-U6 shCD4 or pSilencer 2.1-U6 shGag by using electroporation (Amaxa; Lonza, Switzerland) or lipofection (Lipofectamine LTX, Invitrogen), respectively, according to the manufacturer's protocol. For ELISA, 5×10^6^ PBMCs were infected (MOI, 2) with H6R28LEP or H6R28LEP shGag; 3 d after infection, cells were cotransfected with 20 ng pGL3 vector for standardization and either 1 µg SIN vector plasmid (pCAG-HIVgp, pCMV-VSV-G-RSV-Rev, or CS-CDF-CG-PRE) by using electroporation.

### ELISA

HIV1 Gag p24 expression in culture supernatants was measured by using the HIV1 p24 Antigen ELISA (ZeptoMetrix, USA) according to the manufacturer's protocol. A firefly luciferase plasmid was used as the normalization control. A luciferase assay system kit (Promega, USA) was used to measure the luciferase activity in each cell pellet for standardization between samples.

### Extraction of DNA and RNA from cells

Infected cells were incubated with lysis buffer (10 mM Tris-HCl [pH 8.0], 100 mM NaCl, 25 mM EDTA, 0.45% NP-40, 0.45% Tween 20, 1 mg/ml proteinase K) at 56°C for 4 h and then heated to 98°C for 5 min to inactivate proteinase K. This lysate was used for DNA analysis.

Total cellular RNA was isolated from uninfected or infected cells by using the RNeasy Mini RNA isolation kit (Qiagen, USA) according to the manufacturer's instructions. Total RNA was eluted from RNeasy Mini columns by using 50 µl RNase-free water and then 1 µg of total RNA was reverse-transcribed with primeScript RT Reagent kit (Takara Bio). For detecting siRNA, RNAs larger than approximately 18 nucleotides were isolated from cells by using the miRNeasy miniKit (Qiagen); isolated RNA was concentrated by using the RNeasy MinElute Cleanup Kit (Qiagen) according to the manufacturer's protocol. Samples (1 µg each) of each RNA were reverse-transcribed and a poly(A) tail added by using the miScript Reverse Transcription Kit (Qiagen).

### Real-time PCR

For the analysis of the copy numbers of wtHHV-6 virus and recombinant HHV-6 viruses, each total DNA from infected PBMCs or CBMCs was isolated as described earlier. TaqMan real-time PCR was performed to quantify viral DNA. PCR probes and primer sets for the HHV-6 U5 gene and EGFP gene (Table S1) were used to analyze wtHHV-6 and recombinant HHV-6 viruses, respectively. Amplification and detection were performed with an Applied Biosystems 7300 Real-Time PCR System (Applied Biosystems) by using Premix Ex Taq (Takara Bio). The PCR conditions were 95°C for 30 s, followed by 45 cycles of 95°C for 5 s and 60°C for 31 s. The data were normalized by using the Human TaqMan Copy Number Reference Assay (Applied Biosystems; Life technologies, USA) for the ribonuclease P gene.

To analyze H6R28LEP mRNA expression in H6R28LEP-infected CBMCs and PBMCs, CBMCs and PBMCs were infected with H6R28LEP (MOI, 2), total RNA was purified from infected cells at 1 and 3 d after infection, and cDNA was produced as described above. TaqMan real-time PCR was performed under the conditions described above for immediate early genes U90 and U94; early genes U41 and U69; and late genes U24, U39, and U48, by using the PCR probes and primers shown in Table S1. Data were normalized according to the mRNA levels of the housekeeping gene glyceraldehyde-3-phosphate dehydrogenase (GAPDH).

siCD4 and siGag were quantified by using the miScript Primer Assay Kit and miScript SYBR Green PCR Kit (both from Qiagen) with the universal primer provided in the kit and specific primers for the CD4 gene or HIV1 Gag gene (Table S1). In an ABI 7300 Real-Time PCR System, amplification started with an initial denaturation step at 95°C for 15 min, followed by 40 cycles of 94°C for 15 s, 55°C for 30 s, and 70°C for 30 s, after which the plate was read. Data were normalized according to the mRNA levels of GAPDH.

To evaluate the viability of infected cells, infected and uninfected PBMCs (5×10^5^ each) were harvested every 3 d after infection, total RNA was isolated and quantified, and corresponding cDNAs were synthesized as described earlier. The mRNA levels of various housekeeping genes (GAPDH, β2 microglobulin [B2M], and hypoxanthine guanine phosphoribosyl transferase 1 [HPRT1]) were amplified by using the Human Housekeeping Gene Primer Set (Takara Bio) according to the manufacturer's instructions.

To evaluate the interferon response, cDNAs were synthesized from PBMCs transduced with H6R28LEP shCD4 or H6R28LEP shGag. The relative expression levels of 2′, 5′-oligoadenylate synthetase 1 (Oas1) [24] and signal transducer and activator of transcription 1 (Stat1) [25] mRNAs in the cells were determined by performing real-time PCR using primers in the IFN Response Watcher Kit (Takara Bio) according to the manufacturer's protocol. Data were normalized according to GAPDH mRNA levels.

### Immunofluorescence

To confirm H6R28LEP protein expression in H6R28LEP-infected CBMCs and PBMCs, infected cells were fixed in cold acetone at 1 and 3 d after infection. U90 protein expression was determined using polyclonal anti-U90 rabbit serum as the primary antibody and Alexa Fluor 594-conjugated donkey anti-rabbit immunoglobulin (Invitrogen) as the secondary antibody. OHV-1 [26], OHV-2 [27], and OHV-3 [28] were used as the primary antibodies for U39, U41, and U48, respectively, and reacted with Alexa Fluor 594-conjugated goat anti-mouse immunoglobulin (Invitrogen) as the secondary antibody, at which point the samples were observed under a fluorescence microscope. The polyclonal anti-U90 rabbit serum was prepared in our laboratory from rabbits immunized with a glutathione S-transferase (GST)-U90 fusion protein purified from E. coli.

### Southern blot analysis

Genomic DNA from H6R28LEP-infected CBMCs and PBMCs was analyzed by southern blot hybridization with pSTY07 [29] used as the probe. This probe is the product of *Pst* I digestion of HHV-6B direct repeat sequences (HHV-6B genome nucleotides 806 to 6328) and can detect concatemer binding sites during HHV-6 proliferation. Genomic DNA was purified from H6R28LEP-infected CBMCs and PBMCs. *Apa* I was the only cleaving restriction enzyme discovered that could clearly differentiate between the concatamer binding sites detected by the pSTY07 probe and other fragments. Samples digested overnight with *Apa* I were used as templates, and Southern hybridization was performed using Hybond-N+ and the AlkPhos Direct Labelling and Detection System with CDP-*Star* (both from Amersham; GE) according to the protocol.

## Results

### Structure of recombinant HHV-6 (H6R28LEP)

**Figure 1:**
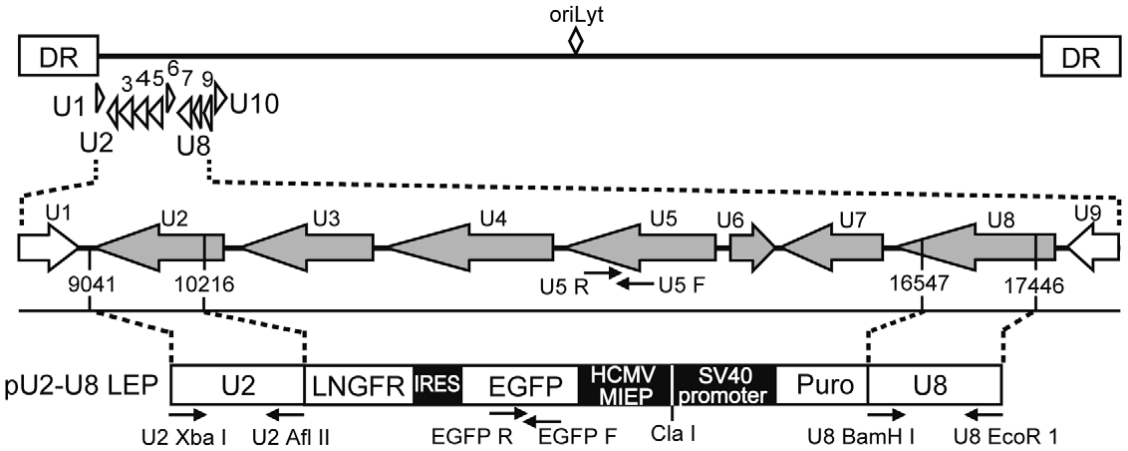
Structure of H6R28LEP. The straight line at the top represents the genome of the HHV-6B HST genome, with the U1 to U10 region expanded below. In the middle, the shaded arrows show the positions from start codon to poly(A) signal of the genes in the U2–U8 gene cluster that was replaced by the EGFP-IRES-LNGFR-Puro cassette. The bottom diagram shows the structure of pU2-U8 LEP. Open boxes represent the low-affinity nerve growth factor receptor (LNGFR), enhanced green fluorescent protein (EGFP), and puromycin-N-acetyl-transferase (Puro) genes; filled boxes represent the internal ribosome entry site (IRES), human cytomegalovirus major immediate early promoter (HCMV MIEP), and simian virus 40 (SV40) promoter. The locations of the PCR primers used in the cloning of the U2 and U8 gene clusters (U2 XbaI, U2 AflII, U8 BamHI, and U8 EcoRI) and the primers for verification of the recombinant and wild-type virus (EGFP F, EGFP R, U5 F, and U5 R) are indicated. DR, direct repeat.

In our previous report, we generated a recombinant HHV-6 by inserting a selectable marker, the puromycin-resistance gene, into wtHHV-6 by homologous recombination [30]. In the present study, we built on this earlier work by using MACS LNGFR microbeads in addition to puromycin treatment to select for transfected cells. In doing so, we created a recombinant HHV-6 virus (H6R28LEP) in a shorter time than previously and were able to concentrate the infected cells. In H6R28LEP, the Puro and LNGFR genes replace the part of HHV-6 genome from the promoter of the U2 gene to the poly(A) signal of the U8 gene (Fig. 1). Using real-time PCR, we confirmed that H6R28LEP lacks expression of the U2–U8 genes (data not shown).

### Proliferative capacity of H6R28LEP

**Figure 2:**
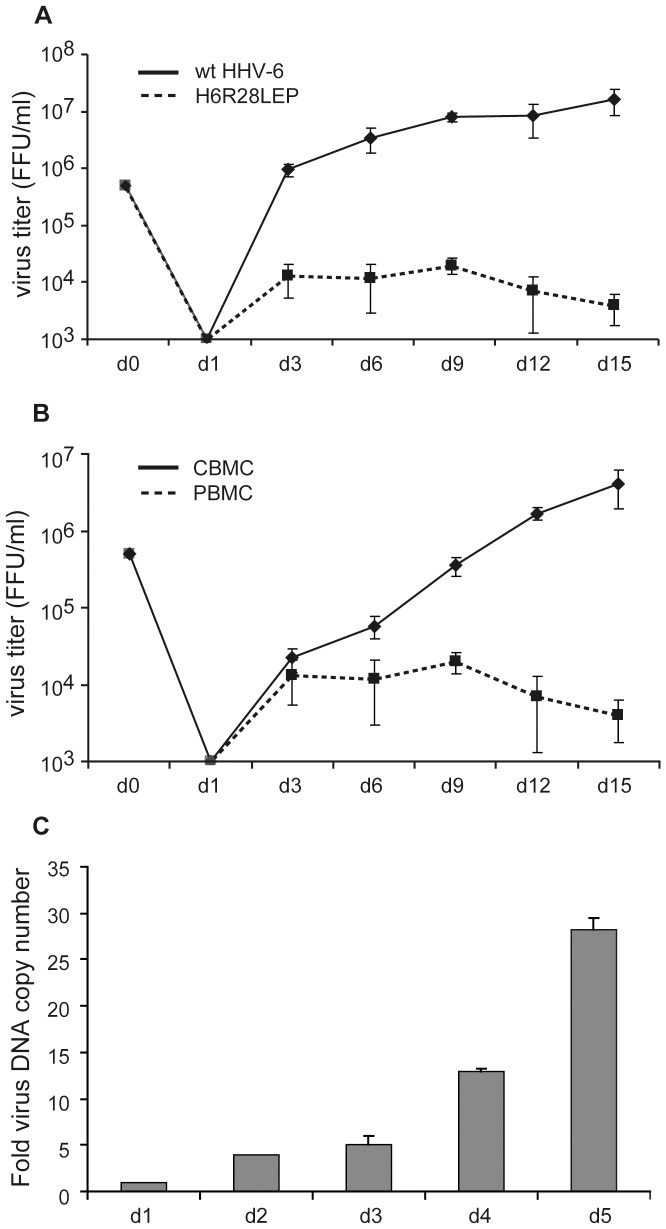
Proliferation of wild-type (wt) HHV-6 and H6R28LEP. **A:** Growth curves for wtHHV-6 and H6R28LEP in peripheral blood mononuclear cells (PBMCs). PBMCs were infected with wtHHV-6 virus or H6R28LEP at a multiplicity of infection (MOI) of 1. Culture supernatant was collected every 3 d, and 1×10^6^ fresh PBMCs were added after each collection. Progeny viruses were titered on MT4 cells by using immunofluroescent assays (wtHHV-6) or by counting EGFP-positive cells (H6R28LEP). Virus titers are indicated in focus-forming units per milliliter (FFU/ml). **B:** Growth curves for H6R28LEP in PBMCs and cord blood mononuclear cells (CBMCs). PBMCs and CBMCs were infected with H6R28LEP, and progeny viruses were titered every 3 d as described for panel A. **C:** Replication of viral DNA in PBMCs. PBMCs were infected with H6R28LEP as described, and infected cells were harvested at the indicated times and frozen at −80°C. Infected cells then were incubated with lysis buffer containing 1 mg/ml proteinase K, and the copy numbers of H6R28LEP in cell lysates were quantified by real-time PCR using primers specific for EGFP. Results are given as mean ±1 standard deviation (*n* = 3).

We infected populations of PHA-stimulated PBMCs with wtHHV-6 and H6R28LEP, collected the culture supernatants 3 to 15 d after the infection, and determined the viral titers of these supernatants. This process revealed that the recombinant virus (H6R28LEP) did not grow in the PBMCs in which wtHHV-6 grew robustly (Fig. 2A). Viral proliferation of H6R28LEP in anti-CD3 antibody-stimulated PBMCs was impaired similarly to that in PHA-stimulated PBMCs (data not shown). When PHA-stimulated CBMCs were infected instead of PBMCs, H6R28LEP produced sufficient titers of infectious viral particles and showed productive infection (Fig. 2B). H6R28LEP and wtHHV-6 demonstrated approximately equivalent proliferation in CBMCs (data not shown). These results indicated that H6R28LEP was a proliferation-defective virus in peripheral blood cells although it behaved as a proliferative virus in the PHA-stimulated CBMCs.

However, levels of H6R28LEP genomic DNA had increased in PBMCs approximately 30-fold by 4 d after infection (Fig. 2C). Therefore, this increase in viral genome DNA copy number was preserved to some extent despite the marked decrease in the production of infectious H6R28LEP viral particles in PBMCs. This increase in viral DNA levels despite decreased production of recombinant viral particles was indicative of efficient expression of the transgene. We consider that this effect is beneficial and supports efficient transgene expression by H6R28LEP. In order to investigate the molecular mechanism of this phenomenon, we examined expression of the H6R28LEP gene and protein in CBMCs and PBMCs. Herpesviruses generally form viral particles after the sequential expression of immediate early, early, and late genes and proteins. Viral gene replication occurs in the early stage and research has shown that late genes are not expressed if viral gene replication does not occur [31]. Therefore, in order to clarify how herpesvirus proliferation is suppressed, knowing at what stage the gene or protein expression is blocked would be useful.

**Figure 3:**
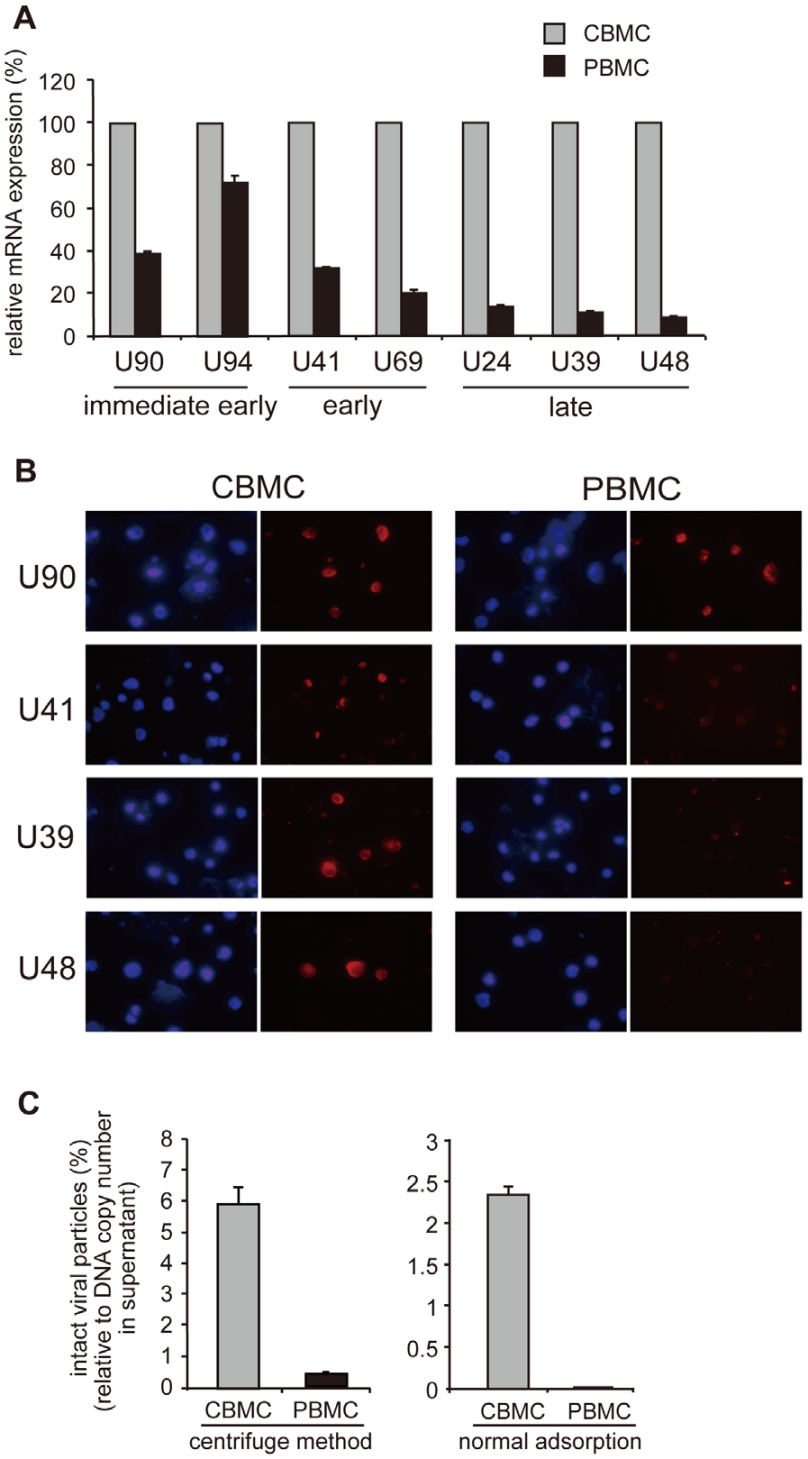
Viral mRNA and protein expression in H6R28LEP-infected PBMCs. **A:** PBMCs and CMBCs were infected with H6R28LEP, total RNA was purified after 24 h, and mRNA expression of immediate early (U90, U94), early (U41, U69), and late (U24, U39, U48) genes was assayed by real-time PCR after RT. The data are shown as mRNA expression volumes in PBMCs relative to those in CBMCs. Data are given as mean ±1 standard deviation (*n* = 3). **B:** At 24 h after infection, H6R28LEP-infected CBMCs or PBMCs were fixed in cold acetone and expression of the viral proteins U90, U39, U41, and U48 was observed using an indirect immunofluorescent assay. **C:** Proportion of H6R28LEP intact virus particles produced in the culture supernatant. CBMCs or PBMCs were infected with H6R28LEP, and real-time PCR was used to quantify the number of H6R28LEP genome DNA copies present in the supernatant after 3 d. The supernatant from each culture was used to infect PBMCs by using centrifugation (left) or the normal adsorption method (right), and the H6R28LEP intact virus particles in the supernatant were assayed. The graph shows the percentage of intact virus particles relative to H6R28LEP genome DNA copy numbers in the supernatant. Data are given as mean ±1 standard deviation (*n* = 3).

For HHV-6, one-step growth is completed and progeny viruses produced in around 24 h. We therefore infected PBMCs with H6R28LEP and investigated mRNA expression of typical immediate early, early, and late genes after 24 h. The results showed that in PBMCs, H6R28LEP mRNA expression was suppressed in the late stage compared with the immediate early stage (Fig. 3A). The results also showed that the expression of late-gene proteins was markedly lower in PBMCs than in CBMCs (Fig. 3B).

To investigate how this incomplete expression of late-gene proteins affects particle formation, we investigated the properties of H6R28LEP particles produced by PBMCs and by CBMCs. We infected CBMCs or PBMCs with H6R28LEP, recovered the viral particles released, and measured the number of genomic DNA copies from these particles as well as the number of viral particles capable of infecting PBMC. The results showed that the percentage of particles (i.e., intact particles) capable of infection was substantially lower for H6R28LEP produced from PBMCs compared with that produced from CBMCs. Infectivity was detected with PBMCs, albeit at a low level, with forced infection of cells by centrifugation. However, H6R28LEP infection of PBMCs hardly occurred with the usual infection method involving viral adsorption (Fig. 3C). These results suggest that intact particles capable of infecting PBMCs are produced when H6R28LEP proliferates in CBMCs, whereas viruses with proliferation defects are produced when H6R28LEP proliferates in PBMCs because intact particles cannot be produced.

**Figure 4:**
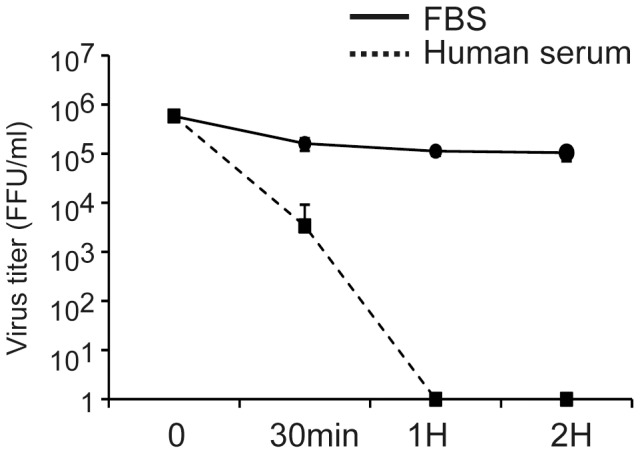
H6R28LEP activity in human serum. H6R28LEP viral solution was mixed with RPMI containing 10% FBS or human serum at a 10∶1 ratio, and the virus titer was assayed after the mixture was left to stand at 37°C for 0, 30, 60, and 120 min. The human serum was sampled from three healthy donors already infected with HHV-6. Data are given as mean ±1 standard deviation.

As most individuals are infected with HHV-6 during their childhood and the virus remains dormant in the body, neutralizing antibodies are likely to be present in the blood. For this reason, HHV-6 vectors can only be used ex vivo to infect target cells. However, this also means there is little risk that contaminating virus could replicate by infecting cells other than the target cells. As the experiments described above suggested changes in the H6R28LEP particle structure, we investigated whether serum from healthy individuals could neutralize H6R28LEP. The results revealed that H6R28LEP is rapidly neutralized by human serum (Fig. 4). We concluded that there is no new risk from contaminating virus.

### Form of the H6R28LEP genome in PBMCs

**Figure 5:**
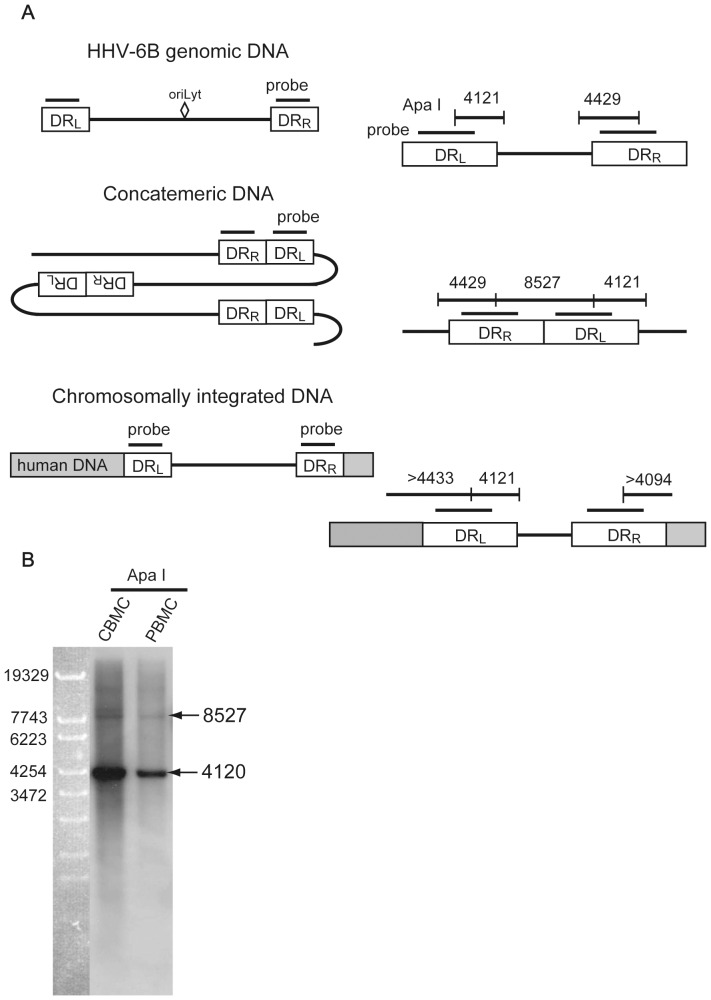
Southern blot hybridization to detect H6R28LEP concatemeric region. Total genomic DNA was purified from H6R28LEP-infected CBMCs and PBMCs after 4 d and digested with the restriction enzyme *Apa* I. **A:** Possible H6R28LEP forms, *Apa* I site locations, probe binding sites, and band sizes as predicted by Southern blot hybridization. Given the impossibility of predicting the gene sequence on the chromosome in the case of chromosomally integrated DNA, we have marked this as >4433 bp and >4094 bp. **B:** In both CBMCs and PBMCs, we detected a 8527 bp band showing concatemeric DNA (viral DNA replicates formed during viral proliferation) and a 4120 bp band showing virion DNA (where the DNA is cleaved into a single genome and packaged into particles).

We used Southern blot hybridization to investigate the form of the H6R28LEP genomic DNA in PBMCs. The results above showed that viral particles could be formed in PBMCs even though H6R28LEP was not intact. This suggests that in PBMCs, H6R28LEP is producing concatemeric DNA, which comprises DNA replicates and is the form assumed by viral DNA when the virus is proliferating. We investigated the ratio of concatemeric DNA to virion DNA, which comprises the viral DNA cleaved into a single genome for packaging into a particle, to see whether the suppression of H6R28LEP proliferation in PBMCs occurs at the gene replication level (Fig. 5A). By using Southern blot hybridization after *Apa* I digestion, we detected an 8527 bp band showing concatemeric DNA (viral DNA replicates formed during viral proliferation) and a 4120 bp band showing virion DNA (single viral DNA genome cleaved for packaging into a particle) in both CBMCs and PBMCs (Fig. 5B). On the basis of the detection sensitivity of Southern hybridization used in this experiment, it is unlikely that we detected plasmid DNA in relatively low numbers or single copies of chromosomally integrated DNA per cell. Therefore, the 8527 bp and 4120 bp bands probably indicate the concatemer binding site and virion DNA, respectively. H6R28LEP viral DNA in PBMCs was also lower than the total amount in CBMCs, although there was no difference in the ratio of concatemeric DNA to virion DNA between PBMCs and CBMCs (Fig. 5B). This indicates that gene replication does not have a major effect on the suppression of H6R28LEP proliferation in PBMCs.

### Infectivity of H6R28LEP

**Figure 6:**
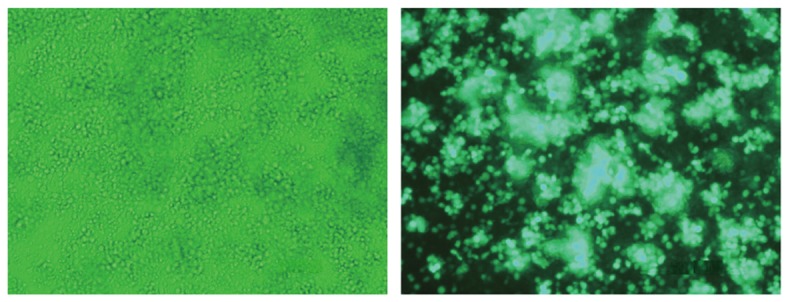
EGFP expression in PBMCs infected with H6R28LEP. PBMCs were infected with H6R28LEP by using the centrifuge method and then were observed under polarizing microscopy (left) and fluorescent microscopy (right) at 4 d after infection.

**Figure 7:**
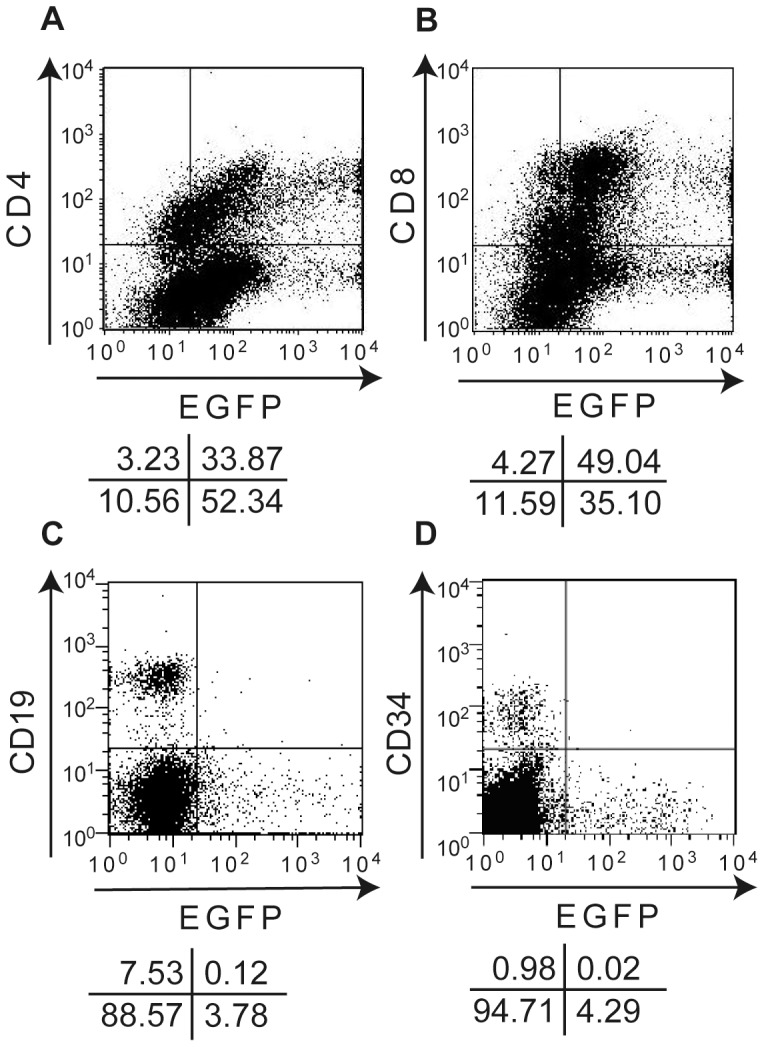
Two-color flow cytometric characterization of surface marker and EGFP expression of PBMCs infected with H6R28LEP. PBMCs were activated by culture with IL-2 and plate-bound anti-CD3 monoclonal antibody for 3 d, infected with H6R28LEP, and underwent fluorescence-activated cell sorting (FACS) analysis at 4 d after infection. **A:** Positivity of CD4 and EGFP in H6R28LEP-infected PBMCs. **B:** Positivity of CD8 and EGFP under the same experimental situation as for panel A. **C:** Positivity of CD19 and EGFP in unstimulated H6R28LEP-infected PBMCs. **D:** Positivity of CD34 and EGFP in unstimulated H6R28LEP-infected CBMCs. The percentage of cells in each quadrant of the FACS profile is shown in the diagram beneath each panel. Results are representative of at least three independent experiments.

According to fluorescence microscopy, PBMCs infected with H6R28LEP remained positive for expression of EGFP for approximately 2 weeks without demonstrating any cytopathic effects. The intensity of the EGFP signal increased daily and was strongest by day 4 after infection (Fig. 6). Flow cytometry revealed that approximately 85% of PBMCs became infected with H6R28LEP (data not shown). Flow cytometry to determine the types of H6R28LEP-infected PBMCs showed that 91.3% of CD4+ T cells and 91.9% of CD8+ T cells were EGFP-positive, but only 1.6% of CD19+ B cells were EGFP-positive (Fig. 7A, B, C). These results show that in PBMCs, H6R28LEP can infect CD4+ and CD8+ T cells at extremely high rates. We also tested infection of CD34+ blood stem cells in umbilical cord blood. The results showed a low infection rate of 2.0%, demonstrating that hardly any infections occurred (Fig. 7D).

### Lack of cytotoxicity after infection with H6R28LEP

**Figure 8:**
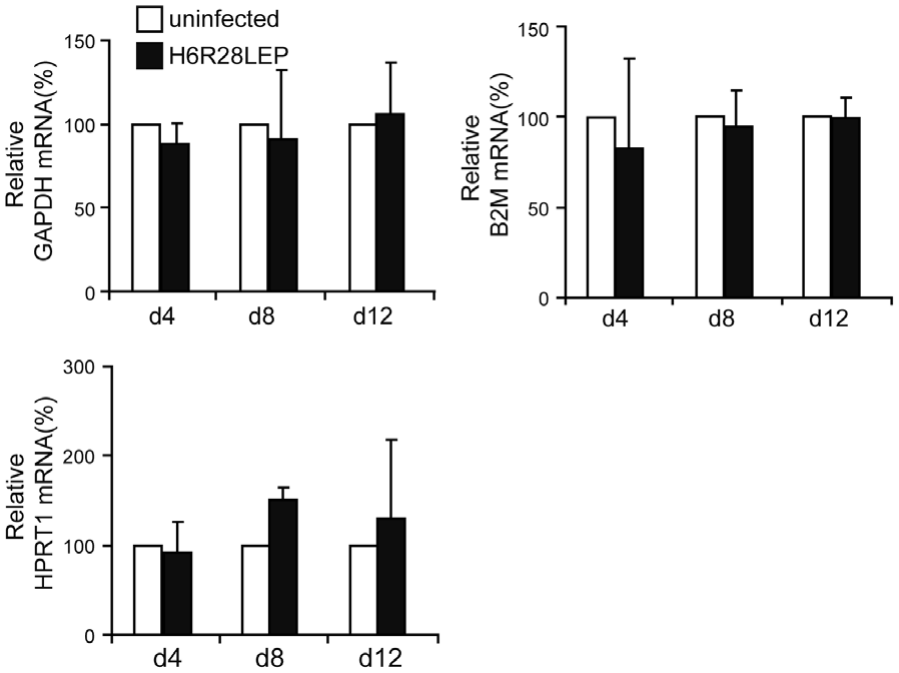
Lack of cytotoxic effects of H6R28LEP as indicated through mRNA expression of housekeeping genes. At the indicated times, infected and uninfected cells (5×10^5^ each) were harvested. Total RNA was isolated and quantified by real-time RT PCR using the Human Housekeeping Gene Primer Set (Takara Bio). Relative mRNA expression levels were compared by using two-tailed Student's *t* tests. Data are given as mean ±1 standard deviation (*n* = 3). GAPDH, glyceraldehydes-30phosphate dehydrogenase; B2M, β2 microglobulin; HPRT1, hypoxanthine guanine phosphoribosyl transferase 1.

We determined the mRNA expression levels of several housekeeping genes (GAPDH, B2M, HPRT1) to examine the effect of infection with H6R28LEP on PBMCs. mRNA expression of these housekeeping genes remained consistent for 12 d after infection (Fig. 8), indicating a lack of effect of H6R28LEP on the function of PBMCs.

### Expression of siRNAs in PBMCs infected with recombinant HHV-6 viruses

**Figure 9:**
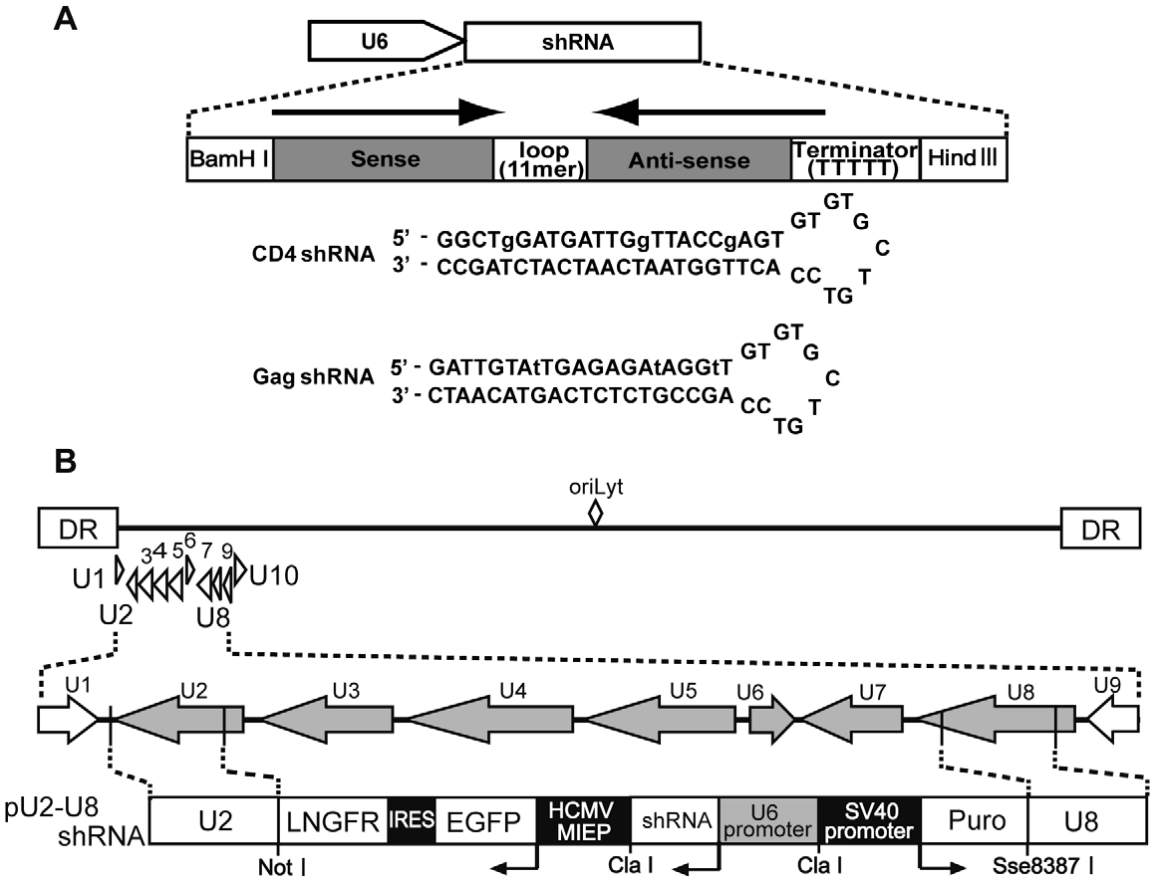
Schematic illustration of short-hairpin RNA (shRNA) constructs and shRNA-expressing H6R28LEP vectors. **A:** Schematic illustration of shRNA constructs. The CD4 shRNA insert contained 22-nt sense and antisense strands, and the Gag shRNA insert contained 20-nt sense and antisense strands. Sense and antisense strands were linked through a standard GTGTGCTGTCC loop structure used in mammalian cells. Two sets of oligonucleotides with the terminator sequence (TTTTT) and overhanging sequences at the 5′ and 3′ ends were synthesized for each shRNA. Lowercase letters indicate the three A-to-G or C-to-T mutations that we inserted into the sense strand to prevent unwanted mutations in *Escherichia coli* cells. **B:** Structure of a H6R28LEP-based vector encoding a U6 promoter-driven shRNA. An shRNA sequence under the control of a U6 promoter was inserted into the *Cla*I site of H6R28LEP. The arrows indicate the transcription initiation site and direction of transcription.

**Figure 10:**
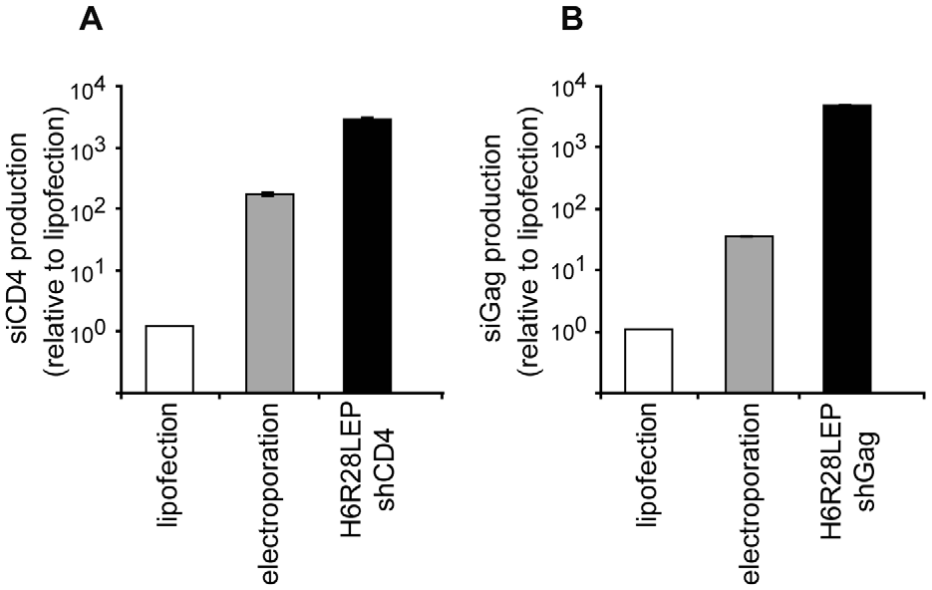
Expression of siRNA determined by real-time RT-PCR. Shown is the relative fold expression of **A:** siCD4 or **B:** siGag in PBMCs. siRNA was transduced through infection of an shRNA-containing H6R28LEP vector or by lipofection or electroporation. Total cellular RNA, including siRNA, was extracted from transduced cells. RNA samples were reverse-transcribed and then amplified by using siRNA-specific primers (siCD4 F and siGag F). Expression of siCD4 and siGag was significantly increased in PBMCs infected with the H6R28LEP-shRNA constructs compared with that after lipofection or electroporation. Data are given as mean ±1 standard deviation (*n* = 2).

To examine the usefulness of siRNA transfected by using H6R28LEP, we generated the virus constructs H6R28LEP shCD4 and H6R28LEP shGag, in which expression of shRNA against the HIV receptor CD4 or the HIV Gag protein, respectively, was driven by using a U6 promoter (Fig. 9). We compared the expression levels of these shRNAs in PBMCs infected by using H6R28LEP shCD4 and H6R28LEP shGag with that from the shRNA expression plasmids transfected into PBMCs by using electroporation or lipofection. The amounts of siCD4 and siGag in the PBMCs infected with the H6R28LEP-based viruses were greater than those in the cells that had been transfected by electroporation or lipofection. Specifically, the amount of siCD4 RNA due to H6R28LEP shCD4 was 4300 times greater than that after lipofection and 30 times greater than that after electroporation. The amount of siGag RNA due to H6R28LEP shGag was 2700 times greater than that after lipofection and 160 times greater than that after electroporation (Fig. 10).

**Figure 11:**
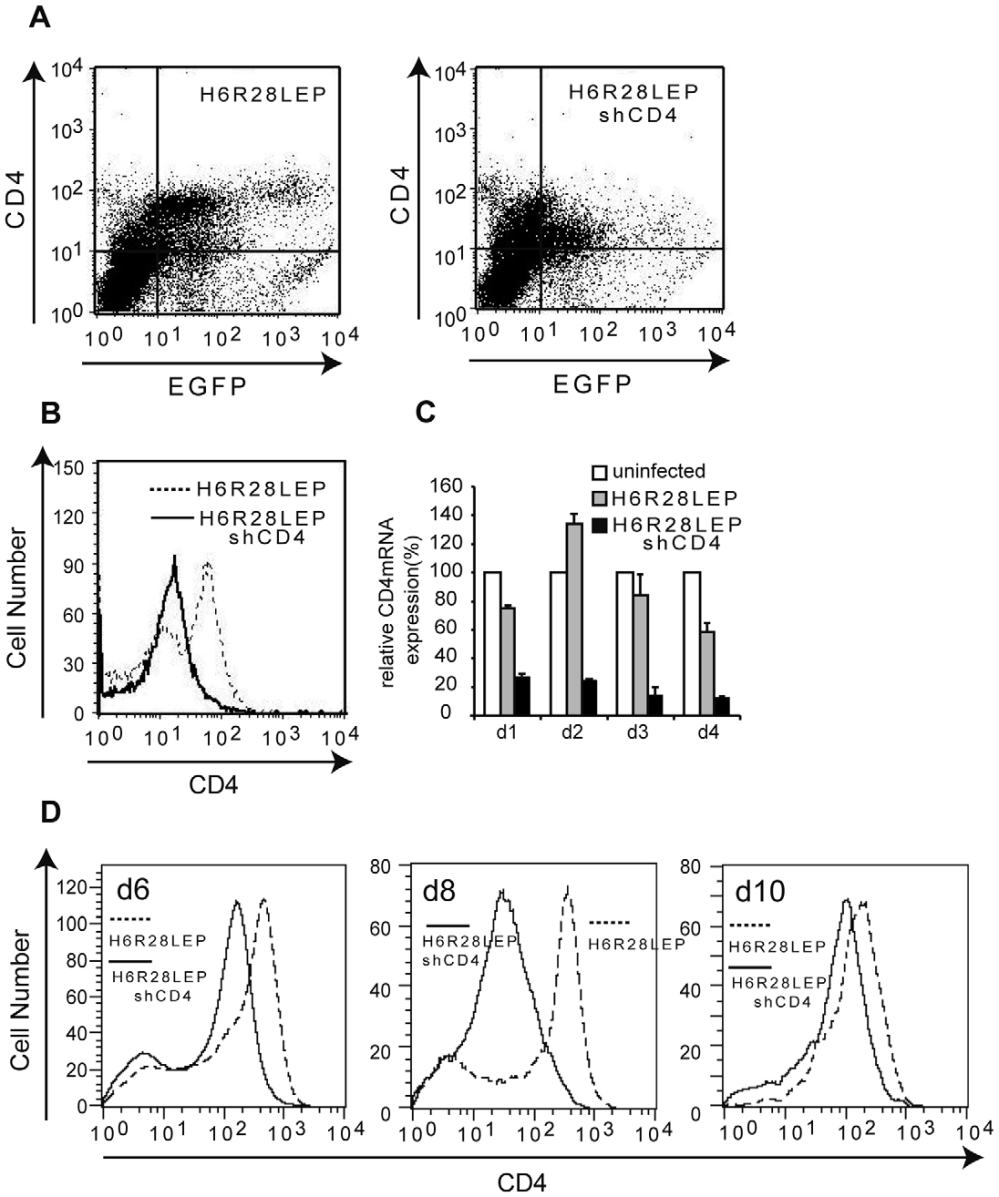
Down-regulation of CD4 protein and mRNA levels in PBMCs infected with H6R28LEP shCD4. PBMCs were cultured on plates coated with 1 µg/ml anti-CD3 antibody for 3 d, and then infected (MOI, 2) with H6R28LEP or H6R28LEP shCD4. **A:** At 4 d after infection, cells underwent FACS analysis for expression of CD4 and EGFP. **B:** Numbers of CD4-positive cells among those infected with H6R28LEP shCD4 (solid line) or H6R28LEP (dashed line) are shown. Results are representative of at least three independent experiments. **C:** Cells were collected at different times after infection, total RNA was isolated, and CD4 mRNA transcripts were quantified by real-time RT-PCR. **D:** Number of CD4-positive cells at 6, 8, and 10 d after infection, as seen in panel B. Data are given as mean ±1 standard deviation (*n* = 3).

Flow cytometry at 4 d after infection showed that, whereas infection rates were similar between the 2 viruses (Fig. 11A), CD4 expression at the cell surface of PMBCs infected with H6R28LEP shCD4 was one tenth that of PBMCs infected with H6R28LEP (Fig. 11B). In addition, CD4 mRNA expression in cells infected with H6R28LEP shCD4 was decreased to less than 10% that of cells infected with H6R28LEP (Fig. 11C). The results confirmed that this effect of H6R28LEP shCD4 continued even at 10 d after infection (Fig. 11D).

**Figure 12:**
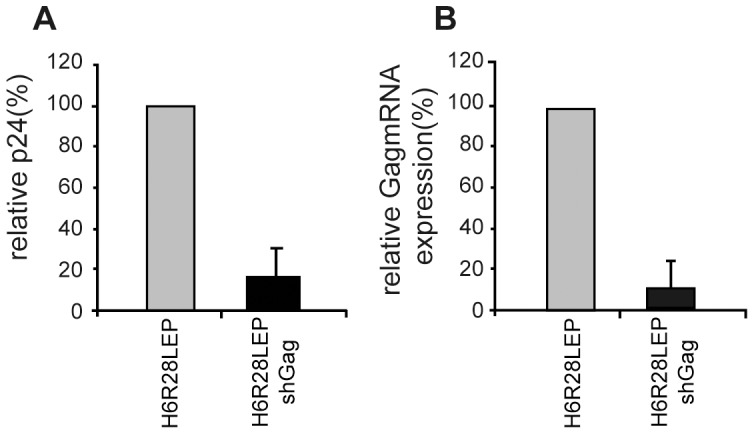
Down-regulation of HIV p24 Gag protein and mRNA levels in PBMCs infected with H6R28LEP shGag. PBMCs infected with H6R28LEP or H6R28LEP shGag were transduced with a self-inactivating HIV vector plasmid by using electroporation. **A:** The p24 level in culture supernatants was determined by ELISA. **B:** Total RNA from transduced PBMCs was isolated, and Gag mRNA expression was quantified by real-time RT PCR. Firefly luciferase expression was used to normalize transfection efficiency, and the expression level of the Gag transcript was normalized against the transfection efficiency. Data are given as mean ±1 standard deviation (*n* = 3).

Furthermore, we examined protein and mRNA levels of HIV p24 Gag in PBMCs infected with H6R28LEP shGag. In this examination, we infected PBMCs with H6R28LEP and H6R28LEP shGag and then used electroporation to transduce the HIV vector plasmid at 3 d after infection. We used ELISA of culture supernatant at 3 d after electroporation to measure levels of p24 protein as a marker for HIV particle production. Cells infected with H6R28LEP shGag yielded less than one fifth of the amount of HIV virus particles produced by H6R28LEP-infected cells (Fig. 12A). In addition, Gag mRNA expression by PBMCs infected with H6R28LEP shGag was less than one tenth of that in the H6R28LEP-infected PBMCs (Fig. 12B). These data correlate with the inhibition of HIV particle production. Therefore, the recombinant HHV-6 vectors produced siRNAs effectively and strongly induced siRNA-associated silencing effects.

### Lack of the interferon response after infection by recombinant HHV-6 vectors

**Figure 13:**
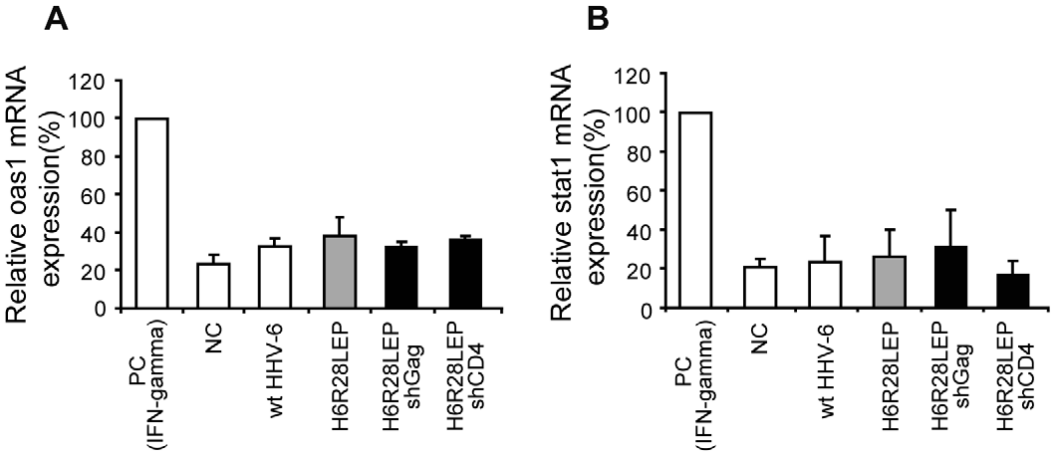
Real-time PCR analysis of OAS1 and STAT1 mRNA levels. **A, B:** PBMCs treated with 100 ng/ml interferon (IFN) γ for 4 d were used as a positive control (PC), and PBMCs treated with buffer alone was used as a negative control (NC). PBMCs were infected (MOI, 0.5) with wtHHV-6 or recombinant HHV-6s, total RNA from transduced PBMCs was isolated at 5 d after infection, and mRNA levels of OAS1 and STAT1 (interferon response genes) were quantified by real-time RT-PCR using IFN Response Watcher (Takara Bio). **A:** OAS1. **B:** STAT1. Data are given as mean ±1 standard deviation (*n* = 3).

It is well known that double-stranded RNA induces an interferon response in mammalian cells, leading to nonspecific inhibition of transcription; in addition, siRNA and shRNA reportedly can induce this interferon response [24] [32]. We examined the mRNA expression of 2 host genes (Oas1 and Stat1) known to be induced through the interferon response [24] [25] to assess whether this response led to the suppression of CD4 synthesis and the anti-HIV effect shown above. The expression of Oas1 and Stat1 did not differ between PBMCs infected with each recombinant virus and uninfected PBMCs (Fig. 13). Therefore, the observed inhibitory effect on CD4 and HIV Gag gene expression likely was due to RNA interference and not to the interferon response.

## Discussion

Because HHV-6 has low virulence [33] and infects CD4^+^ T cells, macrophages, and dendritic cells [14] [15] [34] [35], recombinant HHV-6 vectors have been expected to be useful in gene therapy of HIV infection and cancer. However, the generation of recombinant HHV-6 vectors has lagged behind that of other herpesviruses. Reasons underlying this delay are that HHV-6 has fewer genes than does the closely related human cytomegalovirus (HCMV), and HHV-6 lacks homologs of the HCMV genes that are dispensable for viral replication and therefore easily replaced with transgenes during the construction of recombinant viral vectors [29] [36] [37] [38].

We previously established that a recombinant HHV-6 can be generated by replacing the U3 through U7 genes with those for selectable markers and showed that the resulting virus replicated in PHA-stimulated CBMCs [30]. In the present study, we have shown that the H6R28LEP recombinant HHV-6, which lacks the expression of U2 through U8 genes of wtHHV-6, was unable to replicate in PBMCs (including T cells) but did replicate well in PHA-stimulated CBMCs (Fig. 2A, 2B). Interestingly, H6R28LEP supported replication of viral DNA, albeit at a low level compared with that of wtHHV-6 (Fig. 2C). The U2, U3, U7, and U8 genes all have the β herpesvirus U22 gene family motif [29] [39] [40]. Although detailed functional analyses of the U22 gene family in HHV-6 have not been done, this gene family is known to affect cellular tropism in other β herpesvirus [17] [41]. In addition, U22 family genes are important in viral particle formation [42]. Our experiments on how H6R28LEP proliferation in PBMCs is suppressed suggested that the main causes were incomplete production of late-gene proteins involved in viral particle formation and incomplete formation of infectious particles (Fig. 3B, C). However, the results of Southern hybridization by using HHV-6DR as a probe suggested that active virion DNA was being produced in infected PBMCs owing to the formation of concatemer DNA (Fig. 5).

We surmise that the remarkable decrease of virus production that we observed with H6R28LEP despite its replication of viral genes was due the deletion of the US22 family genes in the U2–U8 region.

Because H6R28LEP multiplied sufficiently in PHA-stimulated CBMCs (Fig. 2B), we believe that removal of the U2–U8 gene cluster from H6R28LEP prevented the virus from efficiently infecting non-fetal T cells. We readily obtained H6R28LEP in sufficient quantity from PHA-stimulated CBMCs. Because CBMCs typically are pathogen-free and unlikely to have problems associated with the induction of malignancy, this recombinant HHV-6 methodology is likely an easy way to decrease the risk of the unexpected side effects that are associated with the cell lines usually used during the production of other replication-defective viruses. HHV-6 actively proliferates in CD4+ T cells. However, the HHV-6 cell receptor has been shown to be CD46, which is present on the surface of nearly all cells [43]. Therefore, the HHV-6 vector H6R28LEP could transduce cells other than CD4+ T cells. Upon investigating H6R28LEP transduction of CD4+ T cells, CD8+ T cells, CD19+ B cells, and CD34+ blood stem cells, we found that H6R28LEP was capable of transducing both CD4+ T cells and CD8+ T cells at extremely high rates (Fig. 7). Because the production of high titers of infectious viral particles is no longer necessary, replication-deficient HHV-6-based vectors may be able to establish abortive infection and thus transduction of cells that typically are not targets of wtHHV-6, such as CD8^+^ T cells.

We predicted that H6R28LEP would have low toxicity because its viral replication was greatly attenuated, and in actuality, transgene expression from H6R28LEP was maintained continuously without impairing cell function for 12 d after infection (Fig. 8). Furthermore, shRNA expression by H6R28LEP continued for around 10 d (Fig. 11D). This suggests that H6R28LEP continues to express functional genes without hindering the T cells for around 10 d. As activated T cells reportedly have a life of only a few days [44], approximately 10 d of gene expression could have some degree of effect on T cell function. The possibility of involving HHV-6 in the treatment of HIV has been supported by not only the fact that HHV-6 infects CD4^+^ T cells, which are target cells of HIV infection, but also by reports that co-infection with HHV-6 and HIV inhibited the increase of HIV [14] [45]. We then sought to assess the potential of shRNA-encoding H6R28LEP as an anti-HIV tool. shRNA-encoding H6R28LEP vector expressed much more shRNA that that obtained after lipofection or electroporation (Fig. 10). In addition, through the synergy of efficient vector-mediated gene transduction and strong shRNA expression, levels of CD4 protein on the surfaces of CD4^+^ T cells were reduced and the expression of Gag molecules in HIV-producing cells was decreased (Fig. 11 and 12). We believe that these results suggest that H6R28LEP might be useful in the treatment of diseases that involve CD4^+^ T cells, such as various immunologic diseases as well as HIV infection. H6R28LEP is rapidly inactivated by human serum (Fig. 4). Therefore, we think there is very little risk of contamination in the blood by virus that has infected target cells ex vivo. This suggests that H6R28LEP is an ideal vector for T-cell transduction ex vivo.

In addition, we found that H6R28LEP did not induce an interferon response, either on its own or through high levels of H6R28LEP-encoded shRNA (Fig. 13), and this property likely will be advantageous when this vector is adapted for clinical use.

Because herpesvirus-based vectors, including HHV-6, can accommodate the insertion of a large transgene into its genome, they potentially will be highly useful in gene therapy applications. HHV-6 is particularly attractive in this context because of its lymphotropism, low pathogenicity, and low risk of inducing malignancy. In addition to its typical infectious pathway, HHV-6 can integrate into host chromosomes [46] [47] [48]. However the specific pathogenicity induced through this integration is unknown [49]. The potential pathogenic risk and beneficial manipulation of HHV-6's ability to integrate into host chromosomes warrant further investigation.

## Supporting Information

Table S1 **Primers and probes used in this study.**
(DOC)
