# Supplementary material for: Novel Gene Therapy Viral Vector Using Non-Oncogenic Lymphotropic Herpesvirus
Source: PLoS One. 2013 Feb 11;8(2):e56027. doi: 10.1371/journal.pone.0056027 (PMC3569415; doi:10.1371/journal.pone.0056027)
Supplement: Table S1 — Primers and probes used in this study. (DOC) [file pone.0056027.s001.doc]

| Name | Sequence (5’ to 3’) |
| --- | --- |
| U2 Afl II | TTACTTAAGTCATCGGGGTCACTATCTTCGCAG |
| U2 Xba I | GCTCTAGACTGCCACGTGAGCGAAAGCATACAC |
| U8 EcoR I | CGGAATTCCTGTGTACCGTCATGGCTTGT |
| U8 BamH I | CCGGATCCGAGTTAATGCATACATGGGAGGCCAGG |
| U5 F | CGAAGAAAAGTAGCACAGGTCTCC |
| U5 R | ACCGTGTCATAAATGCTGAGTTGG |
| U5 probe | FAM-AGGCACCCGTTCCGCCCCAGC-TMRA |
| U24 F | AGCCAAGCCCGTCAAAACTAA |
| U24 R | CTAGCAATGATACATCGTTTGTTGA |
| U24 probe | FAM-ACCGCCTCCGCAGTCATCTCGCT-TMRA |
| U39 F | CTAGGTATCTCACATTCCTCTGTCC |
| U39 R | GAGTGAAACGGTGTGTTATAATCGG |
| U39 probe | FAM-TTGTGAACTCCACGCCTGAGGTTGTCC-TMRA |
| U41 F | TCCACACTACATAAGAAAGCAGC |
| U41 R | TCGTCTTTGACTGAGGATGTGTATA |
| U41 probe | FAM-CAAGTCGCACAGGCATAAGCCAAGTGAT-TMRA |
| U48 F | TGAGCCGCCAATGAACGATA |
| U48 R | CCCTGATGTAATAAATTCCGTGTGA |
| U48 probe | FAM-CCGTACCTGCGGTCAAGCAATTTAACCT-TMRA |
| U69 F | AGTGGTTTACGTCCCTCACAAC |
| U69 R | CTTCGTTGGCCGTCTTTATGG |
| U69 probe | FAM-ACACCTTCCCGTAGGCACCCACTCC-TMRA |
| U90 F | GATGCTCCTTCTTCCACATTACTG |
| U90 R | CCAGAACTGGAAGCCTTTGATC |
| U90 probe | FAM-CATCCCAGAGTCTTCCAACGACATCGCT-TMRA |
| U94 F | AGGGTCTGCCAGCTTTATTCTC |
| U94 R | GATCGATGTCATACTCAATTACTGGA |
| U94 probe | FAM-AATAAAACTGCCGTCCCCACCCCTGC-TMRA |
| CD4 F | CATCCTCTGTCTATTTGAGACTTAGAAA |
| CD4 R | CGACTCCAGCACTTGCTAGTC |
| CD4 probe | FAM-ATCCTACAAGGCTGGCAGTGACAGAACT-TMRA |
| Gag F | GGACCTAGAACGATTCGCAG |
| Gag R | TCTTACTTTTGTTTTGCTCTTCC |
| Gag probe | FAM-GACTGGGACAGCTACAACCATCCCTTCAGAC-TMRA |
| EGFP F | ACTTCAAGATCCGCCACAACAT |
| EGFP R | GGACTGGGTGCTCAGGTAGT |
| EGFP probe | FAM-CGTGCAGCTCGCCGACCACTACC-TMRA |
| siCD4 F | GGCTAGATGATTGATTACCAAGT |
| siGag F | GATTGTACTGAGAGACAGGCT |
